# Surface Vessels Detection and Tracking Method and Datasets with Multi-Source Data Fusion in Real-World Complex Scenarios

**DOI:** 10.3390/s25072179

**Published:** 2025-03-29

**Authors:** Wenbin Huang, Hui Feng, Haixiang Xu, Xu Liu, Jianhua He, Langxiong Gan, Xiaoqian Wang, Shanshan Wang

**Affiliations:** 1 Key Laboratory of High Performance Ship Technology (Wuhan University of Technology), Ministry of Education, Wuhan 430063, China; whut_wenbin_h@163.com (W.H.);; 2 School of Naval Architecture, Ocean and Energy Power Engineering, Wuhan University of Technology, Wuhan 430063, China; 3 School of Computer Science and Electronic Engineering, University of Essex, Colchester CO4 3SQ, UK; 4 School of Navigation, Wuhan University of Technology, Wuhan 430063, China

**Keywords:** intelligent ships, navigation safety, vessel detection and tracking, multi-source sensors, data fusion

## Abstract

Environment sensing plays an important role for the safe autonomous navigation of intelligent ships. However, the inherent limitations of sensors, such as the low frequency of the automatic identification system (AIS), blind zone of the marine radar, and lack of depth information in visible images, make it difficult to achieve accurate sensing with a single modality of sensor data. To overcome this limitation, we propose a new multi-source data fusion framework and technologies that integrate AIS, radar, and visible data. This framework leverages the complementary strengths of these different types of sensors to enhance sensing performance, especially in real complex scenarios where single-modality data are significantly affected by blind zone and adverse weather conditions. We first design a multi-stage detection and tracking method (named MSTrack). By feeding the historical fusion results back to earlier tracking stages, the proposed method identifies and refines potential missing detections from the layered detection and tracking processes of radar and visible images. Then, a cascade association matching method is proposed to realize the association between multi-source trajectories. It first performs pairwise association in a high-accuracy aligned coordinate system, followed by association in a low-accuracy coordinate system and integrated matching between multi-source data. Through these association operations, the method can effectively reduce the association errors caused by measurement noise and projection system errors. Furthermore, we develop the first multi-source fusion dataset for intelligent vessel (WHUT-MSFVessel), and validate our methods. The experimental results show that our multi-source data fusion methods significantly improve the sensing accuracy and identity consistency of tracking, achieving average MOTA scores of 0.872 and 0.938 on the radar and visible images, respectively, and $IDF_{1}$ scores of 0.811 and 0.929. Additionally, the fusion accuracy reaches up to 0.9, which can provide vessels with a comprehensive perception of the navigation environment for safer navigation.

## 1. Introduction

In recent years, while we have witnessed tremendous investment and advances on autonomous driving, there has also been increasing interest in the development and deployment of intelligent vessels with various levels of navigation automation. They hold significant potential to transform traditional shipping with substantial improvements in navigation safety, operational efficiency, and cost effectiveness [1]. Accurate and comprehensive perception of the navigation environment plays a critical role for the intelligent navigation to avoid collisions, supporting efficient and safe navigation planning and decision making. Currently, the common onboard sensors that are equipped in intelligent vessels include AIS, marine radar, and visible cameras. These sensors have been used with deep learning-based models for object detection and tracking to build a comprehensive understanding of the navigation scenes. While extensive research and advances on driving environment perception have been achieved for autonomous driving, the navigation environment and sensing requirements are significantly different from those for autonomous driving. The research works on deep learning models for object detection and tracking are significantly behind those for autonomous driving as well. There are still many challenges to be tackled especially for the complex navigation scenarios. In real-world navigation scenarios, there can be many mixed vessels with various sizes and speeds driving on unstructured waterways. It is very difficult to achieve accurate and reliable sensing with a single type of sensors in these complex maritime environments. For instance, AIS, a system for inter-ship information exchange via radio, can share information such as vessel position, speed, course, and the Maritime Mobile Service Identity (MMSI) [2]. However, AIS is a passive sensing tool that requires other ships within the area to be equipped and to turn on AIS to receive their information. In addition, AIS often suffers from poor real-time performance and reliability. On the other hand, marine radar offers long-range detection and is relatively unaffected by weather, allowing for reliable sensing in rain, fog, and nighttime, making it an indispensable tool for navigation [3]. However, it has many radar blind zones in close-range detection and limited ability to detect small targets. With advancements in deep learning, visible cameras are increasingly used in maritime perception with rich visual details and real-time capabilities [4]. Nevertheless, visible-based sensing typically only provides the position of a target in image coordinates, making it difficult to determine the target’s actual position and speed [5]. Additionally, visible cameras are largely affected by occlusion [6], weather conditions [7,8], and other environmental factors [9,10], which can greatly reduce detection and tracking accuracy in complex scenarios like congested waterways or adverse weather conditions.

Due to the inherent limitations and instability of single-modality perception, an intuitive idea to address these limitations is to integrate multi-source data and exploit their complementarity and redundancy [11]. The integration of multi-source complementary data enhances the informational dimensionality of perception system, enabling more comprehensive characterization of target states. Concurrently, multi-source redundant data improve system robustness, ensuring accurate target perception even in the event of single-source data loss. For example, fusing AIS data with visual detection and tracking results can provide identity and geolocation information for visual targets [12]; combining AIS with radar can effectively detect unauthorized targets without AIS [13]; radar and visual data integration can complement each other’s sensing range. In view of the research gaps and the potentials of multi-source data fusion for maritime vessel perception, we are motivated to develop a novel framework and techniques for the accurate detection and tracking of surface vessel targets. They will integrate multi-source data from AIS and maritime radar and cameras, and leverage the redundancy and complementary information between them for the accurate perception of vessel targets in challenging and complex scenarios.

Firstly, we design a novel multi-stage detection and tracking method (MSTrack), which fundamentally differs from the traditional fusion approaches used in intelligent vessel perception. Conventional methods typically process sensor data independently, performing separate detection and tracking before aggregating the results for unified processing [14,15]. This approach fails to fully exploit the complementary nature of multi-source data during the detection and tracking stages. At the same time, missed detections or tracking errors caused by the inherent limitations of single-modality can negatively affect the overall fusion performance. In contrast, MSTrack introduces a feedback mechanism that leverages historical fusion results to enhance detection and tracking at earlier stage. By integrating fused information back into the detection and tracking modules, our method effectively recovers potential missed detections that occur during the layered processing of radar and visible images. Through this iterative refinement, MSTrack maximizes the benefits of multi-source data fusion, achieving more robust and accurate vessel perception in complex maritime environments.

Secondly, a cascade association matching method is proposed to effectively associate the trajectories from the multi-source data. To achieve association and fusion between multi-source data, kinematics features alignment is essential. However, due to system errors and measurement noise, traditional association methods often suffer from inaccurate alignment and poor robustness, leading to incorrect associations, especially in complex and dense scenarios [12,16]. To overcome the limitations of existing work, we establish the conversion relationships between coordinate systems and select multiple key kinematics features to achieve robust trajectory similarity measurement. Moreover, we propose an innovative multi-source cascade association matching method based on the alignment accuracy. The proposed method fully leverages the characteristics of multi-source data by first performing matching in a high-precision coordinate system, thereby reducing the number of trajectories that need to be associated in a lower-precision coordinate system. Compared to traditional methods, our method significantly reduces the dependence on alignment accuracy during the matching process and ensures that the absence of a single-modality data does not severely impact the overall stability of the fusion system.

Furthermore, to verify the design of our multi-source data fusion techniques, we develop the first multi-source fusion dataset for intelligent vessel (WHUT-MSFVessel), covering a wide range of complex navigation and multiple weather scenarios from the Yangtze River waterways. Unlike autonomous driving, publicly available multi-source fusion datasets remain scarce, which significantly hinders the research and validation on multi-source fusion and autonomous navigation for intelligent vessels.

The main contributions of this paper are as follows:

- We design and implement a novel multi-stage detection and tracking model for intelligent vessels, which feeds the fusion results back to the radar and visible tracking module for the refined detection and tracking of missing targets.
- We design a new cascade association matching method. This approach ensures robust association between multi-source trajectories, even when the kinematics features alignment accuracy is low or certain modality data are missing.
- We develop the first multi-source fusion dataset from the Yangtze River waterway, covering complex navigation and various weather scenarios. The dataset includes synchronized AIS data, radar images, and visible images, filling the gap in multi-source datasets for intelligent sensing and navigation. This dataset has been open sourced for the research community, which can help inspire and promote research on multi-source fusion perception for intelligent navigation in maritime environments.

## 2. Related Works

Surface vessel target detection and tracking is one of the fundamental functions for achieving intelligent navigation in maritime environments. In this section, we will review the recent methods for vessel target detection and tracking. Additionally, we will present the existing association and fusion approaches for multi-source data. Furthermore, we will collect and analyze current perception datasets designed for vessels.

### 2.1. Vessel Detection and Tracking

Target detection based on radar and visible light images shares both similarities and differences, and can be divided into traditional methods and deep learning-based methods. Common traditional radar target detection techniques include the Constant False-Alarm Rate (CFAR) method [17], filtering methods [3], and feature-based approaches [18]. In visible image detection, traditional methods primarily rely on manually designed features [19,20,21] and machine learning classifiers [22,23] to perform the detection tasks. These traditional approaches generally suffer from high complexity, poor generalization, and low detection accuracy.

With the advancement of artificial intelligence, deep learning-based target detection methods, particularly those using deep neural networks, have gradually become mainstream. Popular approaches include the R-CNN series [24,25], YOLO series [26,27,28], and Transformer-based models [29,30]. Among these, YOLO, as a single-stage end-to-end detection network, has gained widespread industrial application due to its superior detection accuracy and real-time performance. In the field of surface vessel target detection, numerous improvements to YOLO have been proposed and extensively researched, with applications for detecting and identifying both visible [31,32] and radar [33,34] vessel targets.

After detecting the positions of targets in an image, the same targets across consecutive frames are associated as the same identity, which is known as multi-object tracking. This approach is also referred as a tracking-by-detection (TBD) framework. It is easy to integrate with various detection algorithms and offers good real-time performance, making it one of the most widely used methods for multi-object tracking [35]. The tracking performance of the TBD framework depends heavily on the accuracy of the detector. However, missed detections in real-world scenarios remain unavoidable. A key research challenge is how to achieve stable tracking or the re-identification of targets despite missed detections.

A classical solution is to use Kalman filtering to predict historical trajectories [36] and employ the Hungarian algorithm for associating targets across frames, like SORT [37], DeepSORT [38], byteTrack [39] and so on. The prediction results from Kalman filtering can mitigate the effects of short-term measurement loss, but as the prediction step length increases, the results may diverge. In surface vessel target tracking, handling missed detections is a significant research focus. Depending on the cause of the missed detections, methods vary for small-scale targets, occluded targets, and motion blur in rain and fog. Although these methods can improve tracking performance to some extent, missed detections in real-world navigation scenarios are often more complex, and the improvements tailored to specific scenes or datasets may not be easily applicable to actual maritime navigation.

### 2.2. Multi-Source Data Fusion

Vessels are equipped with a variety of sensors, each providing unique advantages. By utilizing data fusion techniques, the integration of multi-source data can effectively improve perception accuracy, expand the perception range, and enhance system fault tolerance [40]. The fusion of AIS and radar data, as essential navigation equipment for vessels, has been widely studied [41,42]. AIS provides static information such as MMSI, vessel name, and call sign, as well as dynamic information including vessel position, speed, and heading. Radar, on the other hand, effectively compensates for the real-time limitations of AIS data. Visible image information aligns most closely with human visual perception, but two-dimensional image data lack depth information, making it challenging to assess target size and distance. As a result, the research focus on the fusion of AIS data with visible targets [43,44] is developing rapidly, enabling visible targets to be associated with MMSI information and latitude/longitude coordinates.

To fully leverage the perceptual advantages of various vessel sensors and devices, research on the fusion of AIS, marine radar, and visible camera has gradually gained attention. A spatial–temporal trajectory similarity measurement method has been proposed to address the challenge of multi-source asynchronous trajectory association and fusion, reducing the complexity of trajectory association while ensuring fusion accuracy [45]. Additionally, a multi-sensor fusion system has been developed for vessel detection and tracking on inland waterways, integrating target detection and tracking algorithms with track association methods to improve the consistency and accuracy of vessel motion data [46].

Overall, research on the fusion of AIS, radar and visible data is still relatively limited. The research on association and fusion algorithms remains at an idealized stage, where it assumes that all sensor devices provide stable detection and tracking trajectories. This assumption leads to significant issues with the robustness of the algorithms when applied to real-world, complex scenarios.

### 2.3. Datasets

The rapid development of object detection and tracking in pedestrian and vehicle domains has been greatly facilitated by the availability of numerous public datasets. However, due to the high costs of equipment and the difficulty of data collection, public available datasets for water surface vessel remain extremely limited. Based on the types of data they include, Table 1 summarizes the currently available water surface perception datasets.

Standalone AIS datasets are generally easy to obtain. In addition to the publicly available datasets, AIS data for specific waterways can also be accessed through government maritime agencies or relevant public organizations. Image datasets are dominated by visible data, and most of these datasets are limited to training detection models. Vessel radar datasets primarily consist of synthetic aperture radar (SAR), which differs significantly from the maritime radar used for navigation in terms of imaging principles and application scenarios [54]. In the case of multi-source datasets, the FloW dataset [53] includes maritime radar and visible images, but its focus is on floating waste on the water surface. The FVessel dataset [46] collected and organized synchronized AIS and visible video data for inland waterway surveillance, which can be used for fusion research. However, to date, there are no publicly available vessel datasets that contain synchronized data from AIS, radar, and visible sources.

## 3. Methodology

**Figure 1:**
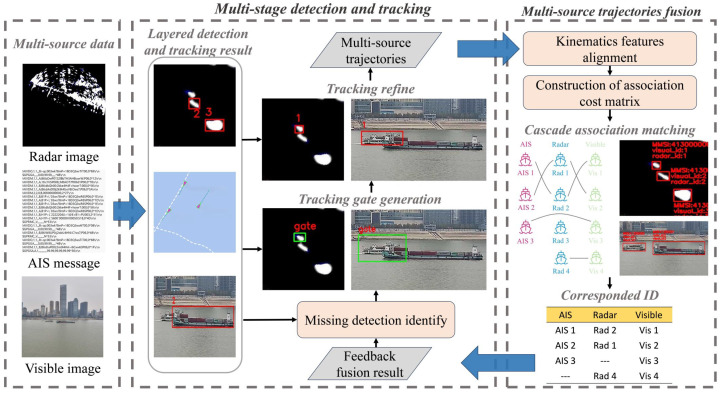
The proposed multi-source data fusion framework for surface vessels detection and tracking, consisting of a multi-stage detection and tracking module and multi-source trajectories fusion module.

The proposed multi-source data fusion framework is illustrated in Figure 1, consisting of two main components. Initially, in the multi-stage detection and tracking module, the multi-source data are processed in a layered way to obtain the vessel detection and tracking results of each data type. Subsequently, the module utilizes the feedback fusion result to identify missing detection and generate tracking gates, within which the missing targets in single-modality layered detection are refined. Then, in the multi-source trajectories fusion module, the kinematic features of the multi-source trajectory are aligned. Unified trajectory features in a common coordinate system are extracted to construct the association cost matrix, aiming to solve the optimal association among multi-source trajectories. Then, through the cascade association matching method, the vessel trajectories will become fused and simultaneously contain the information of MMSI, radar tracking ID, visible tracking ID, latitude, longitude, speed over ground (SOG), and course over ground (COG). The fusion results are output as the final perception results and feedback into the multi-stage detection and tracking module, forming a feedback loop. In order to verify the performance of our algorithm in real scenes, we construct the first multi-weather complex scenes multi-source heterogeneous dataset from the Yangtze River, hoping to provide a benchmark for subsequent research on vessel multi-source sensing methods.

### 3.1. Multi-Stage Detection and Tracking Method

**Figure 2:**
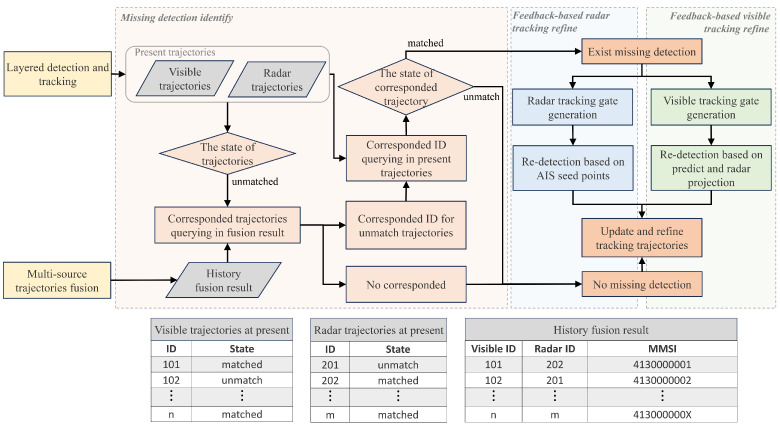
The flowchart of the multi-stage detection and tracking method. The history fusion result feedback from the multi-source trajectories fusion module contains the MMSI, radar ID, and visible ID of the successfully fused target. By querying the fusion data of the previous and the layered detection and tracking results at present, it is determined whether single-modality data have missing detection. Re-detection and tracking with the help of multi-sources complementary data will update the missing trajectories.

Under the TBD tracking framework, missing detections during the detection process can negatively impact the tracking performance. Existing detection algorithms often experience performance degradation and occur missing detections when faced with scenarios involving small-scale objects, occlusions, or environmental challenges like rain, fog, and blur. While some studies have improved detection algorithms to address these specific scenarios, the causes of missing detections in the real world are often diverse and the improvements for specific scenario may struggle to handle the complex environments effectively. Therefore, we propose a multi-stage detection and tracking method (named MSTrack) utilizing multi-source fusion data as illustrated in Figure 2. In this method, we shift the focus away from identifying the specific causes of missing detections. Instead, we leverage historical multi-source fusion data to determine whether missing detections have occurred in the layered detection and tracking processes. Complementary trajectory data from other sensors within the fusion results are then utilized to realize the refine of tracking for missing targets in single-modality perception.

#### 3.1.1. Layered Detection and Tracking

In our method, a layered approach to detection and tracking for each data type serves as the initial step. For AIS data, since the MMSI provides the identity information of the vessel, tracking can be realized by simply preprocessing, such as outliers removal, area restrictions, and life cycles management. For radar and visible images, we select YOLOv8 as the base detection network to ensure rapid and accurate vessel detection, with additional fine-tuning to accommodate the unique characteristics of the data. First, because radar images typically contain small-sized targets, we add an extra small-object detection head to the network to leverage lower-level feature maps, improving the network’s sensitivity to small targets in radar imagery. Then, since AIS data already include vessel class information, we opt for single-class detection in both radar and visible images, removing the classification loss from the network’s loss function to focus the model more on bounding box regression.

Once detection results are obtained, we adopt a TBD framework for vessel tracking, which allows for flexible integration of future method improvements. For radar image targets, which lack stable and distinctive image features, we use the classic simple online and real-time tracking(SORT) [37] algorithm as the baseline. For visible targets, however, we use DeepSORT [38] as the baseline tracking method, as it leverages the rich appearance features in visible images to improve tracking stability. The tracking results can be written as follows:

(1) $$\begin{array}{c} \left\{\begin{array}{c} T_{ais}=\left\{T_{1}^{ais},T_{2}^{ais},\cdots ,T_{i}^{ais}\right\}\\ T_{rad}=\left\{T_{1}^{rad},T_{2}^{rad},\cdots ,T_{j}^{rad}\right\}\\ T_{vis}=\left\{T_{1}^{vis},T_{2}^{vis},\cdots ,T_{k}^{vis}\right\} \end{array}\right. \end{array}$$

where $T_{ais}$, $T_{rad}$ and $T_{vis}$ represent the sets of tracking trajectories for AIS, radar, and visible, respectively. Specifically, $T_{i}^{ais}$ denotes the *i*-th AIS trajectory, while $T_{j}^{rad}$ and $T_{k}^{vis}$ correspond to the *j*-th radar trajectory and the *k*-th visible trajectory.

#### 3.1.2. Missing Detection Identify

First, in layered detection and tracking, there are three possible outcomes for tracking association in each cycle: matched, unmatched detection, and unmatched trajectory. Unmatched trajectory refers to the failure to find an associated detection for a historical trajectory in the current frame. This can indicate that the tracked target has moved out of the perception range, marking the end of the tracking trajectory, or it may be caused by a missing detection.

To distinguish the above cases, we use historical multi-source fusion data to identify the unmatched trajectories, find the missing detection during the tracking process. We first query the ID of the unmatched trajectory from the fusion results of the previous time step. If the trajectory is found in the fusion results and is associated with other sensors trajectories, it indicates that the trajectory has the corresponding multi-sources complementary data. We then search for the corresponding multi-sources trajectory in the current tracking results. If the corresponding trajectory is successfully matched and updated, it confirms that the target is still within the perception range, and mismatch is indeed caused by a missing detection in the single-modality data. The pseudo code of the identify process is shown in Algorithm 1, and the unmatched visible trajectory $T_{UVT}$ and unmatched radar trajectory $T_{URT}$ with missing detection will be output.
**Algorithm 1:** Missing detection identification.
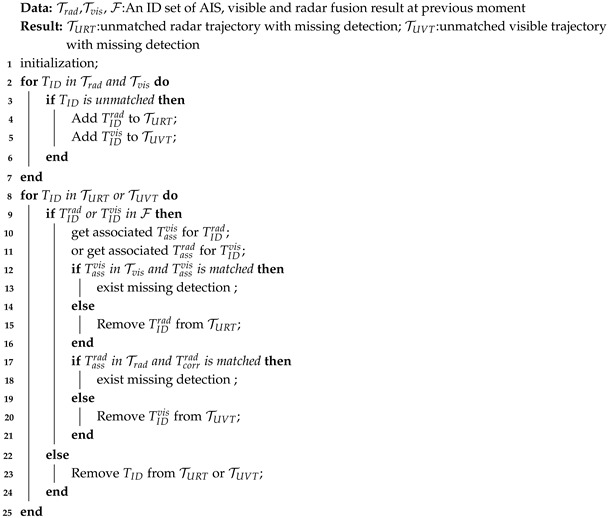


#### 3.1.3. Feedback-Based Radar Tracking Refine

Upon confirming the missing detection for unmatched trajectory, a feedback-based radar tracking refine is applied, utilizing multi-source complementary data. We first generate a box-tracking gate for the missing target, serving as the region proposal. This helps reduce the computational load and prevents the redundant detection of the same target by restricting detection to objects within the tracking gate. Observing the missing detections during the radar tracking process, these issues primarily arise when the echo signal of the target suddenly diminishes on the image. Therefore, using the predicted bounding box generated by the Kalman filter in the tracking process as the tracking gate effectively covers the potential area of the missing target:

(2) $$\begin{array}{c} Bbox_{gate|t}^{rad}=A\cdot Bbox_{det|t-1}^{rad}+B\cdot \mu_{t-1}^{rad} \end{array}$$

where $Bbox_{gate|t}^{rad}$ is the tracking gate of the missing detection at present; $Bbox_{det|t-1}^{rad}$ denotes the radar tracking box before missing; A and B are the state transition matrix and control matrix; and $\mu_{t-1}^{rad}$ denote the control input.

Subsequently, a re-detection process is performed on the targets within the tracking gate. For the radar targets, substantial clutter may still exist within the tracking gate. To address this, we incorporate AIS complementary data as an auxiliary measure. The AIS target’s latitude and longitude are projected to the radar image coordinates to check if any AIS targets are present within the tracking gate.

**Figure 3:**
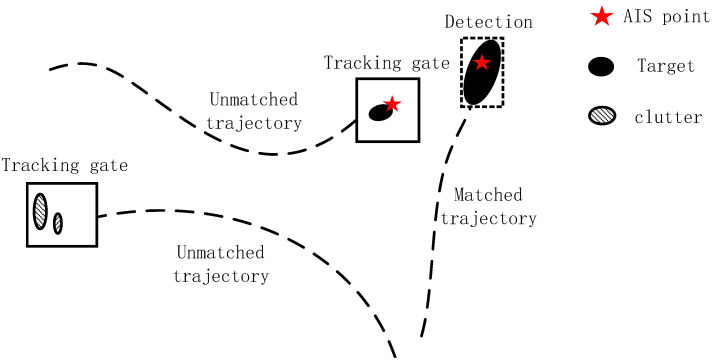
A typical radar scene of re-detection. The AIS data will be used as seed points to help complete the re-detection of radar echoes inside the tracking gate, avoiding clutter interference.

Figure 3 shows a typical radar scene of re-detection. For unmatched trajectories, a box tracking gate is generated by one-step prediction of the Kalman filter. If no AIS points are found within the gate, it is assumed that the gate contains only clutter. While if there exists an AIS point within the gate, this point will be used as a seed point for extracting the connected domain extraction of radar echoes within the gate, and the bounding box of the connected domain will be used as the re-detection value:

(3) $$\left\{\begin{array}{ccc} C & =\{(x,y)|(x,y)\in N(S)\}, & P_{\left(x,y\right)}>T_{p}\\ Bbox_{det}^{rad} & =\left[x_{\min},y_{\min\;},x_{\max},y_{\max\;}\right], & \left(x_{i},y_{i}\right)\in C \end{array}\right.$$

where *S* represents the seed point, N(S) denotes the neighborhood pixels of the seed point *S*, and $P_{\left(x,y\right)}$ indicates the pixel value at coordinates (x,y). $T_{p}$ is the pixel threshold, where pixels exceeding this threshold are identified as target points. *C* denotes the target’s connected region, and $Bbox_{det}^{rad}$ represents the bounding box of the connected region *C*, which will be used to update and refine the unmatched trajectory.

#### 3.1.4. Feedback-Based Visible Tracking Refine

For the refinement of visible tracking, the process can also be divided into two steps: tracking gate generation and the re-detection of missing target. However, due to the high refresh rate of visible data and the different causes of missing detections compared to radar, we adopt a new strategy to refine the tracking results.

**Figure 4:**
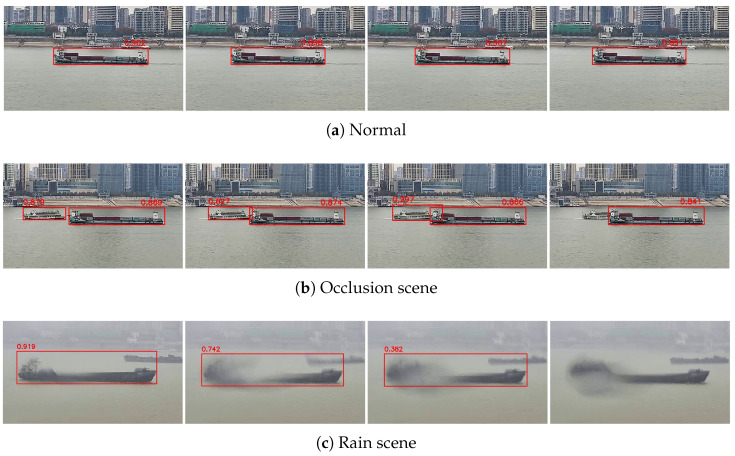
Reduced confidence and missing detection caused by environmental interference. In a normal scene, detection boxes are stable and reliable, but environmental interference causes the target to be unable to recognize and the appearance features change dramatically, which will affect the tracking results.

In the process of visible tracking, environmental factors such as occlusion, rain, or fog often cause a decrease in the detection confidence score. As interference intensifies, this ultimately results in detection miss. Figure 4 illustrates scenarios where the target confidence is affected by environmental disturbances. While some missing target can be recovered by improving the detection algorithm, scenarios with complete occlusion, as shown in Figure 4b, make it difficult to retrieve positional information through detection alone. As for Figure 4c, the blur caused by rain makes it difficult to obtain the accurate boundary of the target, and the appearance feature of the target changes greatly, which will cause interference to the follow-up tracking. Therefore, to achieve the re-detection of missing detection under all scenarios and ensure the stability of detection, we use kinematic prediction combined with a tracking gate to obtain the bounding box for missing targets.

Initially, to obtain more accurate prediction, we further categorize missing detection trajectories and apply different prediction strategies. We select data from the most recent *n* time steps of each visible trajectory to calculate its mean confidence score:

(4) $$\begin{array}{c} m_{conf}=\frac{1}{n}\sum_{i=t-n+1}^{t}conf_{i} \end{array}$$

where *t* denotes the latest time, and $conf_{i}$ represents the confidence score of the detection box at time *i*. If $m_{conf}$ is greater than the threshold $T_{conf}$, the trajectory is considered high confidence; otherwise, it is classified as low confidence. The threshold $T_{conf}$ is set at 0.8.

For high-confidence missing detection trajectories, we use the latest data as the prediction starting point, keeping the prediction step as short as possible to ensure the accuracy. As for low-confidence trajectories, the change of confidence means that the bounding box and appearance feature are not reliable in near-stage. We trace back through the historical trajectory to find the first high-confidence bounding box and perform a linear extrapolation from that starting point to obtain the current predicted value. In this way, we have a more stable predicted bounding box. The prediction’s appearance feature will also inherit from the starting point, trying to avoid the appearance feature being affected by environmental factors. So the visible re-detection value can be gained as

(5) $$\begin{array}{c} \left\{\begin{array}{c} Bbox_{det}^{vis}=\left[\begin{array}{c} x_{s}\\ y_{s}\\ w_{s}\\ h_{s} \end{array}\right]+\left[\begin{array}{c} {\dot{x}}_{s}\\ {\dot{y}}_{s}\\ {\dot{w}}_{s}\\ {\dot{h}}_{s} \end{array}\right]\cdot \left(t_{det}-t_{s}\right)\\ \zeta_{det}=\zeta_{s} \end{array}\right. \end{array}$$

where $x_{s}$ and $y_{s}$ represent the coordinates in the top left corner of the starting point detection box; $w_{s}$ and $h_{s}$ are the height and width of the box; $t_{s}$ denotes the timestamp of the starting point; $t_{det}$ is the timestamp of the re-detection; and ζ indicates the appearance features of the detection result.

The re-detection result $Bbox_{det}^{vis}$ based on kinematic equations extrapolation effectively mitigates the divergence issues commonly encountered in multi-step Kalman predictions. Since the movement of vessels typically follows a uniform and gradually varying pattern, the accuracy of linear extrapolation is generally acceptable in most scenarios. However, to further ensure the reliability of re-detection, we leverage multi-source complementary data to generate tracking gate for the missing target and calibrate the re-detection result. We first project the bounding box of the corresponding radar target onto the visible image to serve as the tracking gate:

(6) $$\begin{array}{c} Bbox_{gate|t}^{vis}=F\cdot Bbox_{det|t}^{rad} \end{array}$$

where $Bbox_{gate|t}^{vis}$ and $Bbox_{det|t}^{rad}$ denote the tracking gate of missing detection and corresponding radar tracking box, respectively; F is the projection matrix from radar image coordinates to visible image coordinates, which are detailed in Section 3.2.1.

If the predicted value $Bbox_{det}^{vis}$ falls within the tracking gate $Bbox_{gate}^{vis}$, it confirms the presence of missing detection, allowing us to use $Bbox_{det}$ as the re-detection and tracking result. The criteria can be given by

(7) $$\begin{array}{c} T_{gate}=max\left(\frac{x_{gate}}{x_{det}},\frac{y_{gate}}{y_{det}},\frac{w_{det}}{w_{gate}},\frac{h_{det}}{h_{gate}}\right) \end{array}$$

where $x_{det}$, $y_{det}$ and $w_{det}$, $h_{det}$ denote the state of $Bbox_{det}^{vis}$, and the variable with the gate subscript is the $Bbox_{gate}^{vis}$ state; a criterion value $T_{gate}$ greater than 1 means that $Bbox_{det}^{vis}$ is within the tracking gate. If $Bbox_{det}^{vis}$ falls outside the tracking gate, we assume that the target has either disappeared or the prediction error is too high, and we discard that detection result.

### 3.2. Multi-Source Trajectories Fusion

**Figure 5:**
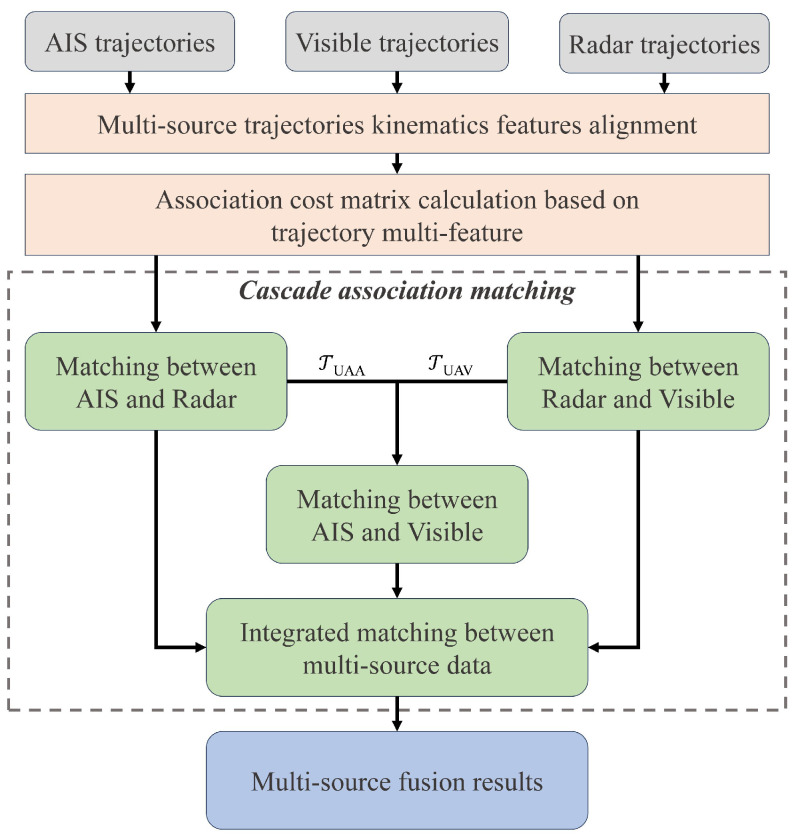
The flowchart of multi-source trajectory fusion. We use the kinematics feature to measure trajectory similarity between multi-source data. And a cascade association matching method is specially designed to reduce the number of trajectories that need to be associated in a lower-precision coordinate system by associating the high-precision aligned data first.

In the proposed fusion framework, the trajectories obtained from the detection and tracking will be fused in the multi-source trajectories fusion module. Figure 5 provides a detailed flowchart within the module. The kinematic feature alignment step establishes temporal and spatial alignment relationships between multi-source trajectories, ensuring a unified metric for subsequent trajectory similarity calculations. For association, we treat the multi-source trajectory association task as a bipartite matching problem. An association cost matrix is constructed using the kinematic features of the trajectories, and the Hungarian algorithm is employed to find the optimal assignment. Additionally, we design a cascaded association matching method, which sequentially associates AIS, radar, and visible trajectories. This method addresses the challenges posed by alignment inaccuracies in real-world scenes. It also ensures that the fusion module can still associate and fuse trajectories data in cases where one trajectory source is missing, enhancing the robustness of the fusion process.

#### 3.2.1. Kinematics Features Alignment

Due to the varying sampling frequencies and spatial coordinates used by different vessel perception devices, kinematics features alignment across AIS, radar, and visible images is essential to measure the similarity between multiple source trajectories. AIS data are characterized by irregular update frequencies and high update delays, typically ranging from 10 s to several minutes. Radar images, constrained by the physical limitations of the antenna scanning speed, generally have an update frequency of 2 s. Visible images, on the other hand, typically refresh at speeds of 25 Hz or higher. Based on these characteristics, we choose to align both AIS and visible images to the radar timestamps. For AIS data, which include information on the vessel’s latitude, longitude, SOG, and COG, we directly apply kinematic equations to perform linear extrapolation, determining the vessel’s position at the radar timestamp:

(8) $$\begin{array}{c} {\mathrm{lat}}_{rt}={\mathrm{lat}}_{last}+\frac{180\cdot \mathrm{SOG}\cdot \Delta t\cdot \cos({\mathrm{COG}}_{\mathrm{rad}})}{\pi \cdot R} \end{array}$$

(9) $$\begin{array}{c} {\mathrm{lon}}_{rt}={\mathrm{lon}}_{last}+\frac{180\cdot \mathrm{SOG}\cdot \Delta t\cdot \cos({\mathrm{COG}}_{\mathrm{rad}})}{\pi \cdot R\cdot \cos(\pi \cdot {\mathrm{lat}}_{last}/180)} \end{array}$$

where ${\mathrm{lat}}_{rt}$ and ${\mathrm{lon}}_{rt}$ denote the latitude and longitude of the AIS extrapolation on the radar timestamp. ${\mathrm{lat}}_{last}$ and ${\mathrm{lon}}_{last}$ are the latitude and longitude of the last AIS data. Δt represents the extrapolation time. R is the radius of the Earth.

Regarding visible images, since vessel movement can be considered a nearly uniform, slow process and the high refresh rate of visible images results in minimal changes between consecutive frames, we simplify the alignment process by selecting the visible image closest to the radar update timestamp as the synchronized data. All data are fused at the radar update timestamps. For non-fusion timestamps in visible data, the detection and tracking results are updated in real time, inheriting the corresponding sensor information from the last fusion timestamp.

Due to the differences in data sources and imaging principles, AIS, radar images, and visible images use different coordinate systems. AIS data are represented in latitude and longitude coordinates, while radar and visible light images are expressed in pixel coordinates. Furthermore, radar images can be considered bird’s-eye views in geodetic coordinates, where visible images are generated through projection transformations based on the pinhole camera model. As a result, longitude and latitude coordinates can be transformed into radar pixels through affine transformations:

(10) $$\begin{array}{c} \left[\begin{array}{c} x_{r}\\ y_{r}\\ 1 \end{array}\right]=Z\left[\begin{array}{cc} R & T\\ 0^{T} & 1 \end{array}\right]\left[\begin{array}{c} lon\\ lat\\ 1 \end{array}\right] \end{array}$$

where $x_{r}$ and $y_{r}$ represent the coordinates of longitude and latitude point on the radar image; Z are rotation and transfer matrices; R and T are the rotation matrix and transfer matrix, respectively.

For visible images, we assume the camera to be an ideal pinhole imaging model, and transform the longitude and latitude coordinates as follows:

(11) $$\begin{array}{cc} \left[\begin{array}{c} x_{v}\\ y_{v}\\ 1 \end{array}\right] & =Z\left[\begin{array}{cccc} \frac{f}{d_{x}} & 0 & u_{0} & 0\\ 0 & \frac{f}{d_{y}} & v_{0} & 0\\ 0 & 0 & 1 & 0 \end{array}\right]\left[\begin{array}{cc} R & T\\ 0^{T} & 1 \end{array}\right]\left[\begin{array}{c} lon\\ lat\\ h\\ 1 \end{array}\right]\\ \; & =ZM\left[\begin{array}{cc} R & T\\ 0^{T} & 1 \end{array}\right]\left[\begin{array}{c} lon\\ lat\\ 1\\ 1 \end{array}\right] \end{array}$$

where M denote the intrinsic matrix of the visible camera, *f* in the matrix is the focal distance of the camera, $d_{x}$ and $d_{y}$ represent the physical size of each pixel; $u_{0}$ and $v_{0}$ are the coordinates of the central pixel of the image; and *h* represents the position height of the target since the vessels all navigate on the water surface. *h* is set to be constant.

#### 3.2.2. Association Cost Calculation

In this study, we take the association between multi-source trajectories as a bipartite matching problem and adopt the Hungarian algorithm to determine the optimal matches. The key aspect of this process lies in the construction of the association cost matrix. Ideally, this can be achieved by aligning all data into a common coordinate system and building the cost matrix based on positional and distance information between targets. However, due to the presence of systematic errors and measurement errors, a cost matrix relying solely on positional information often fails to meet the accuracy requirements for association and fusion in real-world complex scenes.

Therefore, in order to better measure the association cost between multi-source trajectories, we use kinematic parameters *S*=$\left(x,y,\vec{v},a,t,l\right)$ to comprehensively describe the target state, where x,y denotes the target’s position coordinates, $\vec{v}$ represents the target’s velocity vector, *a* is the target’s azimuth, *t* is the start time when the target enters the fusion range, and *l* is the trajectory length. During association and fusion, data from two sensors are first associated, with the association cost calculated as follows:

(12) $$\begin{array}{c} c_{i,j}^{\left(A,B\right)}=\left\{\begin{array}{c} +\infty ,if\left|l_{i}-l_{j}\right|>l_{limit}\;or\left|t_{i}-t_{j}\right|>t_{limit}\\ \frac{k\cdot \sqrt{{\left(x_{i}-x_{j}\right)}^{2}+{\left(y_{i}-y_{j}\right)}^{2}}+\left(1-k\right)\left|a_{i}-a_{j}\right|+\left|\left|\vec{v_{i}}\right|-\left|\vec{v_{j}}\right|\right|}{\cos(\vec{v_{i}},\vec{v_{j}})},otherwise \end{array}\right. \end{array}$$

The formula defines $c_{i,j}^{\left(A,B\right)}$ as the association cost between the *i*-th trajectory from sensor *A* and the *j*-th trajectory from sensor *B*. If the time difference or trajectory length difference between the two trajectories exceeds a predefined threshold, they are deemed unlikely to be associated, and the association cost is set to infinity. The parameters $x,y,\vec{v}$ and *a* are normalized during the cost calculation. The weight coefficient *k* is set to a default value of 0.5. The term $\cos(\vec{v_{i}},\vec{v_{j}})$ represents the cosine distance of the velocity vectors:

(13) $$\begin{array}{c} \cos\left(\vec{v_{i}},\vec{v_{j}}\right)=\left(\frac{\vec{v_{i}}\cdot \vec{v_{i}}}{\left|\vec{v_{i}}\right|\cdot \left|\vec{v_{j}}\right|}+1+\varepsilon \right)/2\in \left(0,1\right] \end{array}$$

where ε is a small constant added to prevent the cosine distance from becoming zero in the denominator. As the angle between two velocity vectors is larger, the cosine distance approaches zero, leading to a higher association cost. This effectively distinguishes targets that are spatially close but have differing headings.

#### 3.2.3. Cascade Association Matching

Based on Equation (12), the association cost matrix between multi-source trajectories can be constructed. However, since the fusion module involves multiple coordinate systems, the association cost across different coordinate systems may vary due to the alignment accuracy. For instance, AIS targets can achieve relatively precise alignment with radar image coordinates through affine transformation, but in visible image coordinates, AIS target alignment can exhibit large errors due to the lack of depth information and simplifications in the camera model. Additionally, in real-world scenes, issues such as missing sensor or trajectories may arise, such as some vessels not activating their AIS. The association and fusion method how to perform under such circumstances is also a critical problem.

To solve the problems mentioned above, we propose a cascade association matching method to achieve accurate and robust fusion among the AIS, radar, and visible trajectories. The cascading association matching method is also shown in Figure 5. In the cascade association matching method, it first establishes pairwise associations between the AIS and radar, as well as between the radar and visible, trajectories. Since visible images lack depth information but can accurately capture the target’s azimuth angle, the weight coefficient *k* is set to 0.2 during the radar and visible matching process. For the unassociated AIS trajectories($T_{UAA}$) and visible trajectories($T_{UAV}$), the AIS trajectories are then projected into the visible image coordinate system for further association. Although the alignment accuracy of AIS targets in the visible image coordinate system is not high, the previous two steps have already filtered out most of the trajectories. The remaining targets are relatively independent, thus reducing the demand for alignment accuracy. Finally, in integrated matching, based on the radar matching result, the AIS and visible trajectories that share the same radar matching ID are considered associated, enabling the cascade matching of the information from all three sources. Thereby, the multi-source data association and fusion is completed.

### 3.3. Datasets

#### 3.3.1. Sensor Setup

As there are no publicly available multi-source perception datasets for surface vessel targets that include synchronized AIS, radar, and visible data across diverse scenes, we develop a multi-sensors platform, including AIS receiver, marine radar, and visible camera, to collect the synchronized data. The collected data are organized into a dataset to validate the performance of the proposed framework and provide an benchmark for future research, which is referred to as WHUT-MSFVessel. The specific parameters of each sensor are as follows:

- The AIS receiver, a Simrad NAIS-500 Class B AIS, captures signals at VHF frequencies of 161.975 MHz and 162.025 MHz.
- The radar, a Simrad HALO20+ marine radar with a detection range of 36 nautical miles, outputs two-dimensional radar images.
- The visible camera, a Hikvision DS-2DE7440IW-A, is equipped with a 1/2.8″ CMOS image sensor. It outputs images at a resolution of 2560 × 1440.

**Figure 6:**
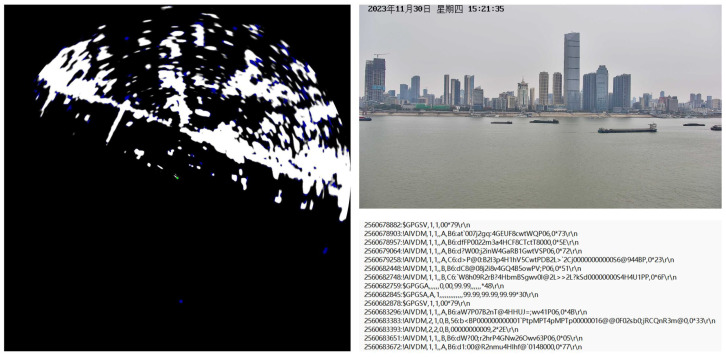
Examples of collected data in WHUT-MSFVessel dataset, including radar images, visible images, and AIS coded messages.

The platform is positioned perpendicular to the Yangtze River channel, with deployment coordinates at [114.306087, 30.563549] and a height of 20 meters above the water surface. The data collection spans 12 months to ensure sufficient diversity in the dataset. The original format of the multi-source data in the dataset is illustrated in Figure 6.

#### 3.3.2. Detail Description

**Figure 7:**
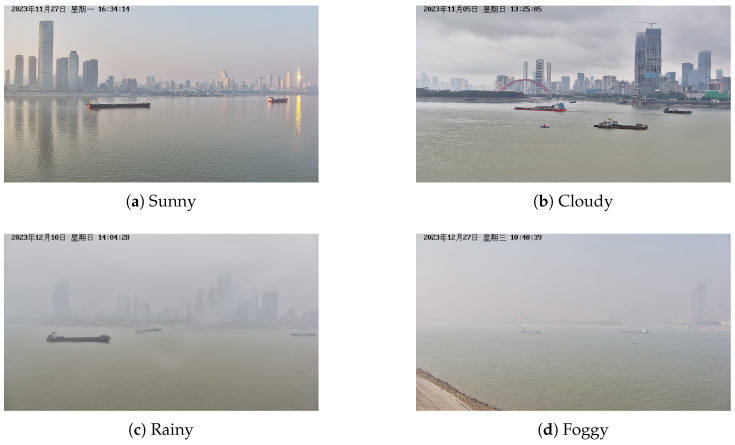
Some samples of visible image representing different weather conditions in WHUT-MSFVessel dataset, including sunny, cloudy, rainy, and foggy.

This study constructs the dataset consisting of 22 scene segments. It covers a variety of weather conditions, such as sunny, cloudy, rainy, and foggy, as shown in Figure 7, to reflect complex real-world situations. All multi-source heterogeneous data are collected synchronously. AIS data are stored in their raw coded format as text formats. Radar and visible data are provided as a continuous frame, each named with a millisecond-accurate timestamp. Meanwhile, visible data also contain the original video with a refresh frequency of 25 Hz. Table 2 shows more detailed information for each scene, including parameters such as scene duration, the total number of radar and visible targets, the maximum number of targets within the camera view, the number of occluded targets, and weather.

**Figure 8:**
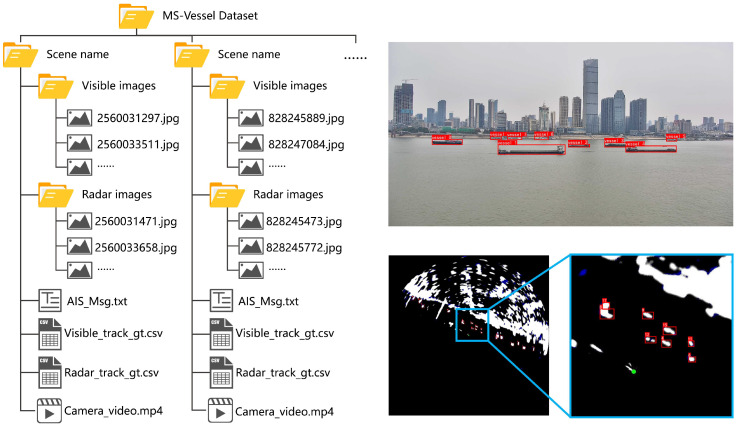
The dataset structure and annotation examples. The data of each scene include AIS text file, synchronized visible and radar images, annotation data stored in csv files, and camera video. During the annotation process, bounding boxes are used to cover vessel targets, and each target is assigned a globally unique tracking ID.

The visible and radar images in WHUT-MSFVessel are labeled by using the professional tools Darklabel to draw a bounding box covering the vessel targets in each frame. Since the class of targets in the radar images is difficult to identify, and the AIS data contain precise ship types, only one class, vessel, is used for labeling. For radar targets that are difficult to distinguish from clutter, professionals with extensive radar using experience will identify and label them by referring to information from adjacent frames to ensure annotation accuracy. Additionally, each vessel entity will be assigned a globally unique ID to facilitate identification in tracking tasks. All annotation data are stored in the same CSV file format as the MOT dataset [55]. The dataset structure and annotation examples are shown in Figure 8.

As the first publicly available multi-source fusion dataset specifically designed for intelligent maritime navigation, WHUT-MSFVessel addresses the longstanding data gap in this field, establishing a standardized benchmark for future research. Distinguished from existing datasets, WHUT-MSFVessel integrates data from the three most common sensing data in vessel navigation: AIS, marine radar, and visible camera. It achieves spatiotemporal synchronization for heterogeneous data collection under real-world navigation conditions, which has not been seen in previous studies. Additionally, all data are annotated in a unified format, allowing flexible applications in various perception tasks such as detection, tracking, and fusion. Furthermore, the dataset encompasses diverse weather conditions and navigation scenes, enabling the comprehensive evaluation of algorithm performance and robustness under different environments.

## 4. Experimental Results

### 4.1. Implementation Details

To validate the detection and tracking performance of MSTrack and the overall fusion performance of the proposed framework, we conducted experiments on the WHUT-MSFVessel dataset.

First, the WHUT-MSFVessel dataset was manually divided, with scene01 to scene05 designated as the testing set, specifically for evaluating the performance of detection, tracking, and fusion. The remaining scenes were used as the training set for network model training. This division was made because the images in our dataset are continuous video frames. If a random sampling method were used to divide the training and testing sets, the images in two sets would be similar, thus failing to objectively reflect the real detection and tracking performance. Meanwhile, scenes 01–05 encompass all typical scenarios in the dataset, covering various weather conditions (sunny, cloudy, rainy, and foggy) as well as different vessel densities. This selection ensures a comprehensive evaluation of the proposed method’s real-world performance across diverse scenarios.

In the detection and tracking module, YOLOv8 was used as a basic detector for the TBD tracking framework. Training on the WHUT-MSFVessel dataset was performed on an NVIDIA A100 GPU. The training utilized SGD as the optimizer, with a batch size of 16 and an initial learning rate of 0.01. The visible data were trained for 200 epochs, while the radar data required only 100 epochs to achieve satisfactory convergence. Additionally, we fine-tuned and retrained the re-identification network within the tracking algorithm to better adapt to the scale and features of vessel targets. The track buffer length in the tracking algorithm is also a key parameter, which will determine the number of frames to retain the unmatched trajectory. Due to the high refresh rate and slow vessel motion during visible tracking, the track buffer length of all algorithms was set to 300. During radar tracking, the value was set to 5.

**Figure 9:**
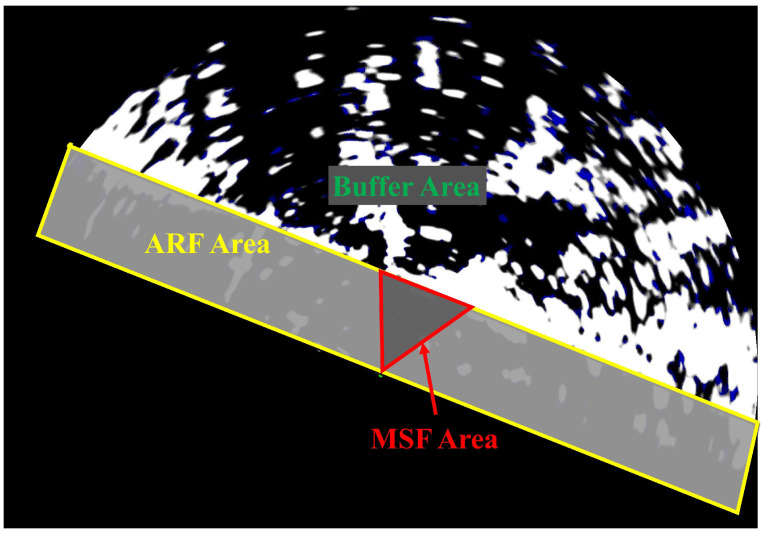
Different areas in the multi-source perception scene. AIS data in the buffer area are cached before entering the fusion areas. In the ARF area, the AIS and radar trajectories are fused. The MSF area is the camera’s field of view; only this area contains AIS, radar, and visible data at the same time, so the multi-source fusion is carried out in this area.

During the processing of multi-source heterogeneous data, the AIS data cover targets within approximately 10 km of the collection platform, while the radar detection radius is set to 5 km, and the horizontal field of view of the visible camera is 54.3°. Based on these parameters, the multi-source perception scene is divided into three areas: the buffer area, the AIS–radar fusion (ARF) area, and the multi-source fusion (MSF) area as illustrated in Figure 9. Different preprocessing and fusion strategies are applied to each area.

First of all, although the radar scan image is a circular area, the data collection area is located within an inland waterway, resulting in significant irrelevant land areas in the images. To address this, a rectangular region is defined to segment the waterway area as the fusion area. Areas outside the rectangle are designated as the buffer area, with only AIS data being managed inside it. The buffer area exists to address the low update frequency of AIS data, ensuring that targets entering the fusion area immediately possess AIS trajectories that can be used for alignment and fusion. Within the fusion area, further division is based on the camera’s field of view, represented by a triangular region in Figure 9. This creates the ARF area and the MSF area. The triangular region is designated as the MSF area, where all three data types coexist, and multi-source fusion is performed using the cascade matching method described in Section 3.2.2. In the ARF area, only the association and fusion of AIS and radar trajectories are carried out. The fusion frequency for all data is synchronized with the radar refresh rate.

### 4.2. Evaluation Metric

For evaluating the performance of detection and tracking, we use the multiple object tracking accuracy (MOTA) [56], identification $F_{1}$ score ($IDF_{1}$) [57], mostly tracked target (MT) and mostly lost targets (ML) as the evaluation metric. MOTA provides a comprehensive measure of the impact of different types of errors during tracking. The calculation formula is as follows:

(14) $$\begin{array}{c} MOTA=1-\frac{\sum (FN_{t}+FP_{t}+IDSw_{t})}{\sum GT_{t}} \end{array}$$

where FP and FN represent the numbers of false positives and false negatives in the detection results; IDSw indicates the number of identity switches during tracking, representing tracking-related errors; GT denotes the total number of ground truth targets; and *t* means the t-th frame during the tracking process. A MOTA score closer to 1 indicates fewer detection and tracking errors, thereby reflecting better tracking performance.

$IDF_{1}$ focuses on the accuracy of target ID in tracking results, assessing the algorithm’s performance in maintaining identity consistency. $IDF_{1}$ is calculated by evaluating the ID matching between predicted and ground truth targets, combining the identification precision (IDP) and identification recall (IDR) into a harmonic mean:

(15) $$\begin{array}{c} IDP=\frac{ID_{TP}}{ID_{TP}+ID_{FP}} \end{array}$$

(16) $$\begin{array}{c} IDR=\frac{ID_{TP}}{ID_{TP}+ID_{FN}} \end{array}$$

(17) $$\begin{array}{c} IDF_{1}=\frac{2\times IDP\times IDR}{IDP+IDR} \end{array}$$

where $ID_{TP}$, $ID_{FP}$, and $ID_{FN}$ represent the targets that are correctly detected with the correct ID, correctly detected but with an incorrect ID, and missed targets without an ID, respectively.

MT refers to the mostly tracked target, the ratio of ground-truth trajectories that are covered by a track result for at least 80% of their respective life span. Conversely, ML indicates the mostly lost targets, referring to tracking result that are covered for at most 20% of their life span. These metrics reflect the method ability to maintain continuous tracking of targets.

To quantitatively assess the performance of multi-source data fusion, in this paper, we propose the following evaluation metrics:

- True Positive Fusion (TPF): AIS, radar and visible targets are correctly associated and fused.
- Part Positive Fusion (PPF): The fusion information correctly associates data from one sensor, but the other sensor’s information is incorrectly associated.
- False Positive Fusion (FPF): Incorrect association between radar tracking IDs, visible tracking IDs, and MMSI.
- False Negative Fusion (FNF): The target fails to be successfully fused.

Based on these metrics, fusion accuracy and fusion error rate are used to provide a comprehensive description of the fusion results:

(18) $$\begin{array}{c} Accuracy=\frac{TPF+0.5\cdot PPF}{GT} \end{array}$$

(19) $$\begin{array}{ccc} Error & =\frac{0.5\cdot PPF+FPF+FNF}{GT} & =1-Accuracy \end{array}$$

### 4.3. Detection and Tracking Results

In this section, to validate the effectiveness of our proposed tracking method, MSTrack, we conducted experiments across multiple scenes and compared its performance with the baseline and several state-of-the-art TBD tracking methods, including SORT [37], DeepSORT [38], Bytetrack [39], and BotSORT [58]. To ensure a fair comparison, all tracking methods were tested using the same initial detection results, eliminating the influence of the detector.

#### 4.3.1. Radar Detection and Tracking Results

Table 3 presents the quantitative evaluation results of the above mentioned tracking methods across multiple scenes in radar images. Our method achieves average MOTA and $IDF_{1}$ scores of 0.872 and 0.811, respectively, representing improvements of 23.9% and 28.3% compared to the baseline SORT. When compared to other advanced algorithms such as ByteTrack and BotSORT, our method also demonstrates significant advantages, with average MOTA improvements of 20.6% and 19.6%, respectively, and average $IDF_{1}$ improvements of 20.0% and 18.9%. Meanwhile, the IDSw and ML metrics in MSTrack are significantly lower than with other methods, and most of the trajectories are successfully tracked. These results demonstrate that our MSTrack significantly improves the continuity of target tracking.

**Figure 10:**
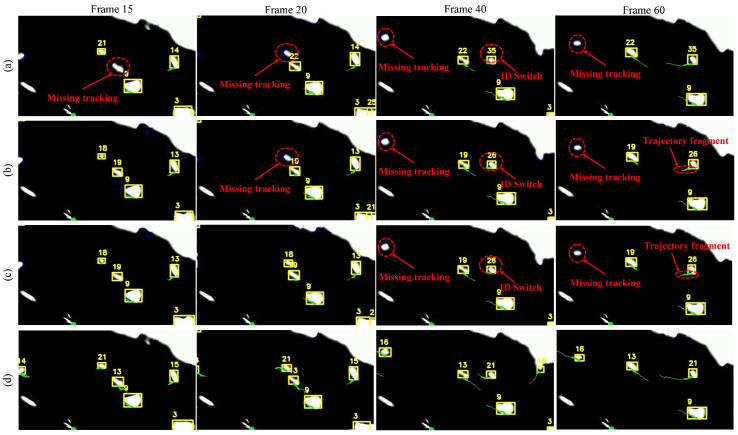
Radar tracking result compared with different methods. Errors in the tracking are marked by the red dotted circle. From top to bottom, the methods shown are (**a**) SORT, (**b**) Bytetrack, (**c**) BotSORT, and (**d**) our MSTrack.

Figure 10 illustrates the tracking results of vessel targets selected from four frames within a sequence of 45 consecutive radar images. The vessel tracking targets in the image are marked in yellow boxes, with the tracking ID attached on the upper left corner of the detection box, and the green line indicates the movement trajectories of the targets. It can be clearly observed that, in the SORT method, target 21 was mis-tracked at frame 20 and was re-tracked at frame 40 but with an ID switch to 35. At frame 40, SORT, ByteTrack, and BotSORT all encountered similar errors, which stemmed from missing detections during single-radar-based detection, limiting the performance of the tracking algorithms. Additionally, in frame 60, the trajectory of target 26 in both ByteTrack and BotSORT, although free of identity switches, incurred trajectory fragment. In contrast, our MSTrack method leverages multi-sensor fused data to continue tracking missed targets, effectively overcoming the limitations imposed by the detector and providing more stable tracking with complete target trajectories.

**Figure 11:**
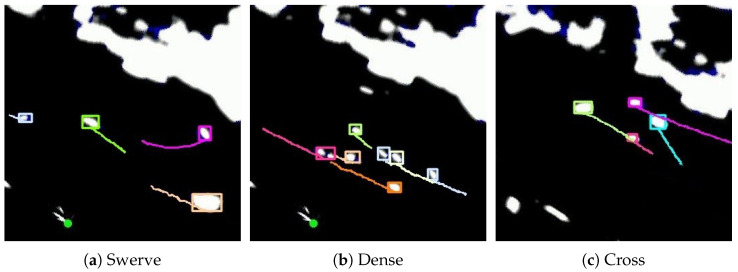
Some samples of radar tracking in different scenarios, including swerve, dense, and cross.

Figure 11 shows the tracking performance of MSTrack across different scenarios. It can be observed that regardless of maneuvering, dense or trajectory crossing scenarios, the proposed method successfully achieves the continuous and stable tracking of all vessel targets in the images.

#### 4.3.2. Visible Detection and Tracking Results

For visible images, our method also achieves superior performance across evaluation metrics as shown in Table 4. The average MOTA and $IDF_{1}$ scores reach 0.938 and 0.929. Notably, the IDR metric, which represents recall, shows a significant improvement with an average score of 0.92, marking a 25.5% increase compared to the baseline DeepSORT. This indicates that our method effectively mitigates issues such as missing detections and tracking failures, thereby improving the continuity of tracking trajectories. In certain scenes, such as scene03, ByteTrack and BotSORT exhibit similar performance, which we attribute to the limitations of single-modality data detection, creating bottlenecks for TBD tracking methods. In contrast, our MSTrack method leverages multi-source data to further enhance tracking performance, achieving MOTA and $IDF_{1}$ scores of 0.981 and 0.99 in this scene.

**Figure 12:**
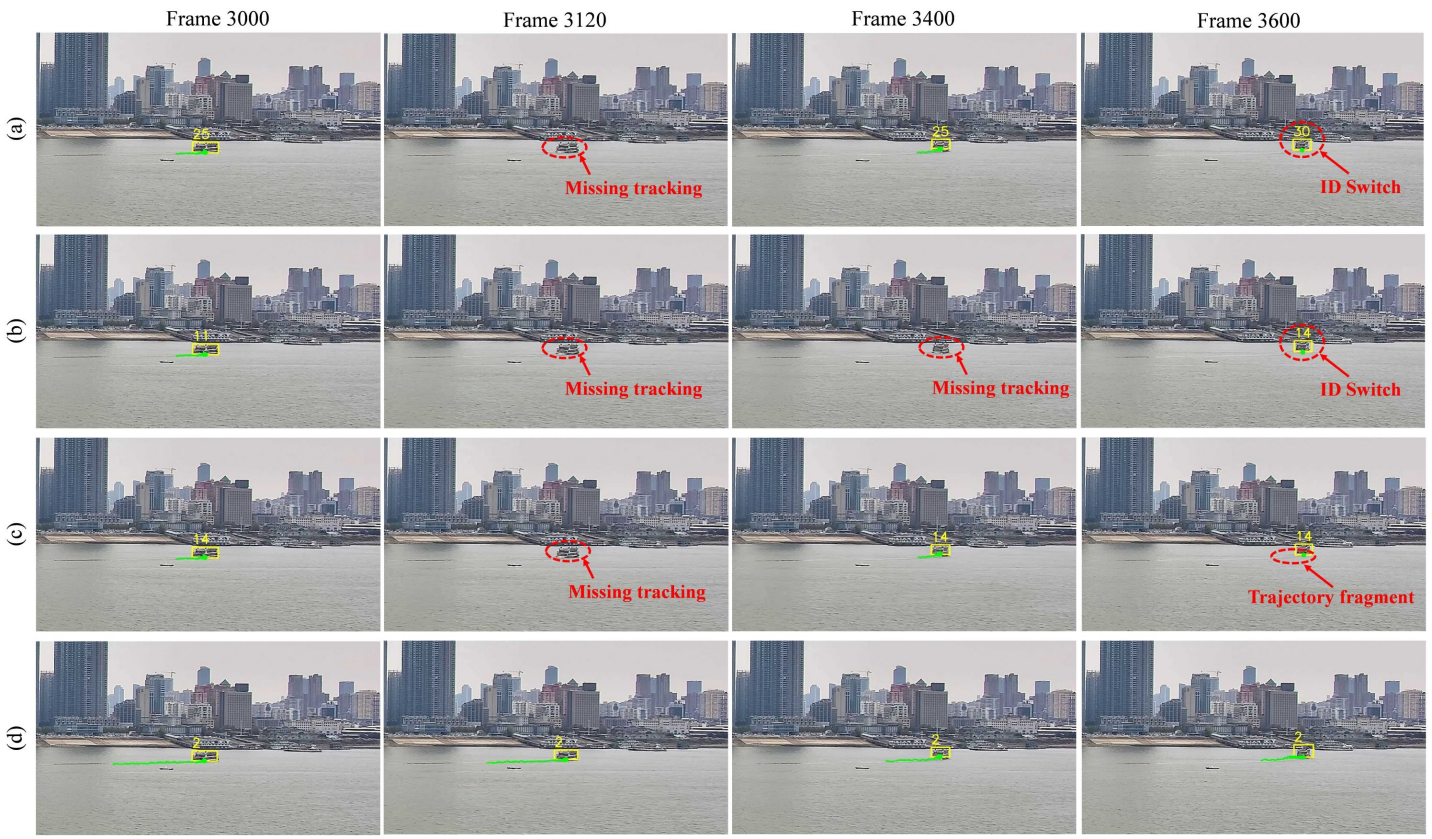
Visible tracking result compared with different methods. Errors in the tracking are marked by the red dotted circle. From top to bottom, the methods shown are (**a**) DeepSORT, (**b**) Bytetrack, (**c**) BotSORT, and (**d**) our MSTrack.

Figure 12 shows the tracking performance on a small target in visible images over a continuous period of 600 frames. The target, with dimensions of approximately 60 × 35 pixels, occupies about 0.05% of the total image area. Its small size and lack of appearance features lead to unstable detection results. As a result, DeepSORT, ByteTrack, and BotSORT all fail to track the target at frame 3120. At frame 3600, DeepSORT and ByteTrack experience identity switches, while BotSORT incurs trajectory fragment. In contrast, our MSTrack method, utilizing feedback from multi-sensor data, successfully re-detects and tracks the missing small target, maintaining the completeness of tracking trajectories and the consistency of target identities.

**Figure 13:**
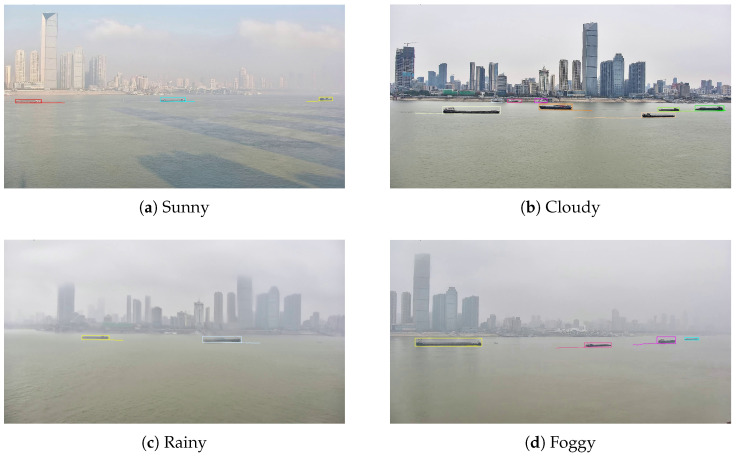
Some samples of visible tracking in different weather conditions, including sunny, cloudy, rainy, and foggy.

Figure 13 intuitively presents the visible tracking performance of MSTrack under various weather conditions. The vessel target trajectories in the images are smooth and continuous, demonstrating stable tracking even in challenging conditions such as rain and fog, effectively validating the robustness of MSTrack.

### 4.4. Multi-Source Data Fusion

The multi-source fusion results provide the rich complementary data of vessels, ensuring the safety of the navigation. Table 5 shows the quantitative analysis of the association and fusion results. It demonstrates that our association method can achieve multi-source data fusion at an accuracy of over 0.9 in most cases. Among the evaluation metrics, FNF has the greatest impact on our fusion accuracy because the visible trajectory needs to undergo an initialization process when the target enters the MSF area, which leads to an increase in the number of FNF.

**Figure 14:**
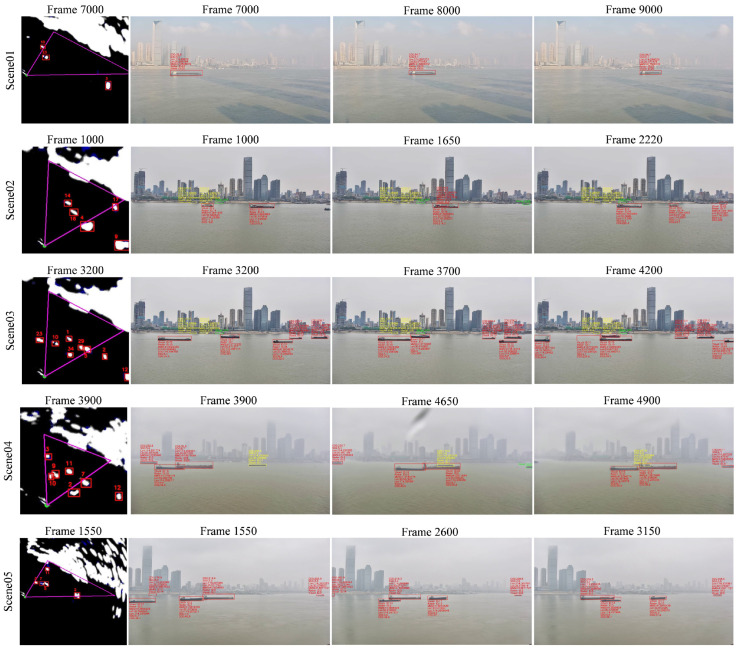
The fusion result in the main view of visible images in different scenes.

Due to the intuitive nature of visible image, Figure 14 uses it as the primary viewpoint to illustrate the effects of multi-source data fusion. The triangular area in the radar image denotes the visible field of view, within which the AIS, radar, and visible data are fused. For targets with the successful fusion of AIS, radar, and visible data, red bounding boxes are used in the visible image. Partially fused targets and those that failed to fuse are marked with yellow and green boxes, respectively.

As shown in Figure 14, vessels in motion generally maintain a stable association across multi-source data. Using the associated radar tracking ID, we can quickly link the same target between visible and radar images. Additionally, the fused AIS data provide the visible targets with latitude, longitude, speed, and other navigational information, aiding in maritime situational awareness. In scene02, for instance, three vessels docked along the shore have radar echoes that blend with the land, causing them to go undetected in the radar image. However, due to our cascading matching structure, visible vessels 4 and 5 still fuse with AIS data, marked in yellow boxes. Visible vessel 6 in scene02, which is in a docked state with inactive AIS, lacks fusion information and is therefore marked with only a green box to indicate the visible detection result. In scene04, a vessel that has not activated AIS is marked with a yellow box. Despite this, the target is successfully associated with its corresponding radar trajectory, obtaining complementary information such as latitude, longitude, and speed. The fusion results above clearly demonstrate the robustness of the proposed cascading association matching method and fusion framework. Even when the partial trajectory is missing, our method can still flexibly achieve the association and fusion of multi-source data, ensuring information complementarity. Moreover, both quantitative and qualitative results indicate that our fusion framework not only performs effectively in simpler scenes like scene01 but also accurately fuses multi-source trajectories in more complex and dense scenes such as scenes 02–05.

### 4.5. Running Time Analysis

The real-time performance of the proposed method is a crucial metric, which determines its feasibility for practical engineering. To evaluate this, we analyze the processing time of each module under different scenes, including the detection, tracking, fusion, and overall processing time. The detailed results are presented in Table 6.

The results reveal that the detection module accounts for a significant proportion of the overall processing time, but it is relatively stable. Additionally, scenes 02 and 03 contain a larger number of trajectories compared to other validation scenes, leading to higher processing times for the tracking and fusion module, which indicates a positive correlation between the processing time and the number of trajectories. The average and maximum overall processing times are 31.1 ms and 34.8 ms, respectively, which correspond to frame rates of 32 FPS and 29 FPS. These metrics confirm that the proposed method essentially satisfies the requirements for real-time applications.

## 5. Conclusions

In this paper, we propose a novel framework for surface vessel detection and tracking that fuses multi-source heterogeneous data. It was designed to effectively address the limitations of single-modality data in maritime environment perception for intelligent vessels. In the framework, we designed a multi-stage detection and tracking method, which effectively exploits the redundant and multi-source complementary fusion to identify missing detections, and refines the detecting and tracking of the missing targets. Then, we designed a cascade association matching method to improve association accuracy in dense and complex navigation scenarios, which allow for robust association between multi-source trajectories. To validate our method, we created the first multi-source fusion dataset in the maritime domain, laying a benchmark for future research. Experiments on typical scenarios in the dataset demonstrated that our algorithm significantly enhances detection and tracking accuracy, with average MOTA scores of 0.872 and 0.938 on the radar and visible images, respectively, and IDF1 scores of 0.811 and 0.929. They can provide more accurate and comprehensive environmental perception for the safe navigation of intelligent vessels. However, the proposed multi-source fusion framework is still based on decision-level fusion and does not fully leverage all pixel-level information from radar and visible images. Our future work will focus on deeper levels of fusion of multi-source data and improving the perception accuracy for intelligent vessels.

## Figures and Tables

**Table 1:** Most cited public accessible datasets for water surface perception compared with our WHUT-MSFVessel dataset.

Datasets	AIS	Radar	Visible
Piraeus AIS [47]	✓		
OpenSARShip [48]		✓	
HRSID [49]		✓	
Seaship [50]			✓
MaSTr1325 [51]			✓
MaSTr1478 [52]			✓
FloW [53]		✓	✓
FVessel [46]	✓		✓
WHUT-MSFVessel (ours)	✓	✓	✓

**Table 2:** Detail of the WHUT-MSFVessel dataset. The “NR”, “NV”, “MN”, and “OC” represent the total number of radar targets, the total number of visible targets, the maximum number of targets in camera view, and the number of occluded targets, respectively.

Scene	Duration	NR	NV	MN	OC	Weather
scene01	09m06s	12	4	3	1	Sunny
scene02	04m20s	25	7	6	3	Cloudy
scene03	08m20s	21	10	9	1	Cloudy
scene04	07m01s	17	6	5	4	Rainy
scene05	07m16s	15	6	5	2	Foggy
scene06	15m20s	32	10	6	4	Sunny
scene07	11m50s	11	5	3	0	Sunny
scene08	14m54s	30	11	6	4	Sunny
scene09	18m38s	17	10	5	3	Sunny
scene10	10m07s	19	8	4	2	Cloudy
scene11	21m22s	12	8	6	0	Cloudy
scene12	13m21s	20	12	6	4	Cloudy
scene13	18m16s	18	7	3	0	Rainy
scene14	14m48s	21	5	4	1	Rainy
scene15	21m23s	25	6	4	2	Rainy
scene16	17m02s	18	7	3	2	Rainy
scene17	23m12s	33	9	6	3	Rainy
scene18	30m17s	19	6	2	2	Foggy
scene19	21m45s	15	5	3	0	Foggy
scene20	19m24s	20	9	5	2	Foggy
scene21	31m36s	27	9	6	0	Foggy
scene22	41m07s	31	12	5	4	Foggy

**Table 3:** Quantitative evaluation results of various radar tracking methods for comparison in different scenes. The comparative tracking methods include SORT [37], Bytetrack [39], and BotSORT [58].

Scene	Method	MOTA↑	IDP↑	IDR↑	IDF1↑	IDSw↓	MT↑	ML↓
scece01	SORT	0.601	0.726	0.573	0.640	15	7	2
	Bytetrack	0.688	0.734	0.572	0.643	14	8	1
	BotSORT	0.691	0.736	0.574	0.645	14	8	1
	MSTrack	**0.830**	**0.776**	**0.724**	**0.749**	**7**	**10**	**0**
scece02	SORT	0.795	0.864	0.717	0.784	14	16	2
	Bytetrack	0.802	0.866	0.743	0.801	12	18	2
	BotSORT	0.802	0.897	0.769	0.828	11	18	2
	MSTrack	**0.912**	**0.891**	**0.886**	**0.888**	**5**	**23**	**1**
scene03	SORT	0.739	0.683	0.521	0.591	51	12	1
	Bytetrack	0.758	0.792	0.631	0.703	24	14	1
	BotSORT	0.762	0.796	0.634	0.706	**23**	14	1
	MSTrack	**0.896**	**0.798**	**0.739**	**0.767**	**23**	**19**	**0**
scene04	SORT	0.716	0.655	0.508	0.572	30	12	2
	Bytetrack	0.701	0.712	0.530	0.607	23	13	2
	BotSORT	0.700	0.711	0.529	0.607	21	13	1
	MSTrack	**0.869**	**0.855**	**0.807**	**0.830**	**11**	**17**	**0**
scene05	SORT	0.727	0.728	0.568	0.638	12	10	2
	Bytetrack	0.703	0.807	0.588	0.680	13	10	2
	BotSORT	0.732	0.812	0.597	0.688	12	10	2
	MSTrack	**0.875**	**0.869**	**0.879**	**0.874**	**6**	**14**	**0**
Average	SORT	0.704	0.719	0.565	0.632	122	57	9
	Bytetrack	0.723	0.774	0.601	0.676	86	63	8
	BotSORT	0.729	0.779	0.607	0.682	81	63	7
	MSTrack	**0.872**	**0.828**	**0.794**	**0.811**	**52**	83	1

**Table 4:** Quantitative evaluation results of various visible tracking methods for comparison in different scenes. The comparative tracking methods include DeepSORT [38], Bytetrack [39], and BotSORT [58].

Scene	Method	MOTA↑	IDP↑	IDR↑	IDF1↑	IDSw↓	MT↑	ML↓
scece01	DeepSORT	0.846	0.855	0.742	0.795	10	3	**0**
	Bytetrack	0.844	0.982	0.838	0.904	6	**4**	**0**
	BotSORT	0.885	**0.987**	0.855	0.917	4	**4**	**0**
	MSTrack	**0.941**	0.979	**0.907**	**0.942**	**3**	**4**	**0**
scece02	DeepSORT	0.906	0.868	0.810	0.838	14	5	1
	Bytetrack	0.906	0.875	0.813	0.843	12	5	**0**
	BotSORT	0.908	0.890	0.827	0.858	7	5	**0**
	MSTrack	**0.935**	**0.964**	**0.947**	**0.956**	**5**	**6**	**0**
scene03	DeepSORT	0.935	0.947	0.945	0.946	13	9	1
	Bytetrack	0.98	0.962	0.959	0.961	6	9	1
	BotSORT	0.979	0.963	0.960	0.961	4	9	1
	MSTrack	**0.981**	**0.986**	**0.994**	**0.990**	**1**	**10**	**0**
scene04	DeepSORT	0.848	0.619	0.569	0.593	28	5	**0**
	Bytetrack	0.858	0.762	0.696	0.728	17	5	**0**
	BotSORT	0.870	0.778	0.710	0.742	14	**6**	**0**
	MSTrack	**0.914**	**0.895**	**0.882**	**0.889**	**4**	**6**	**0**
scene05	DeepSORT	0.833	0.678	0.593	0.633	17	3	2
	Bytetrack	0.835	0.846	0.738	0.788	9	4	1
	BotSORT	0.836	0.792	0.691	0.738	9	4	1
	MSTrack	**0.911**	**0.889**	**0.871**	**0.880**	**2**	**5**	**0**
Average	DeepSORT	0.882	0.796	0.733	0.763	82	25	4
	Bytetrack	0.884	0.894	0.814	0.852	50	27	2
	BotSORT	0.897	0.889	0.815	0.850	38	28	2
	MSTrack	**0.938**	**0.939**	**0.920**	**0.929**	**15**	**31**	**0**

**Table 5:** Quantitative evaluation results of multi-source heterogeneous data fusion in different scenes.

Scene	TPF	PPF	FPF	FNF	Accuracy
scene01	263	0	4	12	0.943
scene02	559	6	2	49	0.917
scene03	1254	27	15	89	0.904
scene04	807	5	5	60	0.920
scene05	598	9	6	69	0.883

**Table 6:** Processing time of different modules in multiple scenes, including detection, tracking, fusion and overall process (unit: ms).

Scene	Trajectory Num	Detection	Tracking	Fusion	Overall
scene01	16	16.6	2.8	2.4	24.5
scene02	32	17.1	5.6	5.3	33.2
scene03	31	17.2	5.8	5.9	34.8
scene04	23	16.8	4.2	4.7	31.7
scene05	21	16.7	4.0	4.5	31.2
Average	-	16.9	4.5	4.6	31.1

## Data Availability

Data are contained within the article. The dataset is available at https://github.com/yixiiii/Multi-Source-Fusion-for-Vessels (accessed on 20 February 2025).
